# Supplementary material for: COZOID: contact zone identifier for visual analysis of protein-protein interactions
Source: BMC Bioinformatics. 2018 Apr 6;19:125. doi: 10.1186/s12859-018-2113-6 (PMC5889581; doi:10.1186/s12859-018-2113-6)
Supplement: Supplementary file 2 — Software build. Executable binary file of the software tool. (ZIP 118,784 kb) [file 12859_2018_2113_MOESM2_ESM.zip › cozoid/java/docs/beansbinding-1.2.1-doc.zip!/org/jdesktop/beansbinding/ext/package-summary.html]

org.jdesktop.beansbinding.ext (Beans Binding)


---


|  |  |  |
| --- | --- | --- |
| **Overview** \| **Package** \| Class \| **Use** \| **Tree** \| **Deprecated** \| **Index** \| **Help** | |  |
| **PREV PACKAGE**   **NEXT PACKAGE** | **FRAMES**    **NO FRAMES**     **All Classes** |  |


---

## Package org.jdesktop.beansbinding.ext

Provides support for customizing Beans Binding, and for
determining the synthetic properties available for binding.

**See:**
  
          **Description**

| **Interface Summary** | |
| --- | --- |
| **BeanAdapterProvider** |  |

| **Class Summary** | |
| --- | --- |
| **BeanAdapterFactory** |  |

## Package org.jdesktop.beansbinding.ext Description

Provides support for customizing Beans Binding, and for
determining the synthetic properties available for binding.
Tool vendors and component authors are the expected audience
of this package.

Some `Property` implementations, such as `BeanProperty`
and `ELProperty`, resolve properties of an object at runtime
as per the Java Beans specification. Sometimes, properties of interest aren't
exposed according to this specification (for example, Swing's
`JTextField.text`, `JSlider.value`, `AbstractButton.selected`).
At other times, we may want to extend an object's set of properties
with synthetic properties that may be interesting for the purposes of
binding (for example `selectedElement(s)`) on Swing's `JTable`
and `JList`.

This package provides for these scenarios by allowing you to provide
adapters to adapt these objects and expose such properties as proper
Java Beans properties. This is done by writing one or more
`BeanAdapterProvider`
classes and registering them via the service provider mechanism
(to be implemented) with the `BeanAdapterFactory`.

`Property` implementations can then query the `BeanAdapterFactory`
for adapters to take the place of the source object for the purposes
of property resolution. This is exactly what `BeanProperty` and
`ELProperty` do. In addition, tools can query the factory for
the set of additional properties exposed for a particular class by
the set of loaded providers.

Note: Adapters for many Swing properties are already registered with the
factory. See the swingbinding
package level documentation for details.

---


|  |  |  |
| --- | --- | --- |
| **Overview** \| **Package** \| Class \| **Use** \| **Tree** \| **Deprecated** \| **Index** \| **Help** | |  |
| **PREV PACKAGE**   **NEXT PACKAGE** | **FRAMES**    **NO FRAMES**     **All Classes** |  |


---
